# Shear-Wave Elastography Assessments of Quadriceps Stiffness Changes prior to, during and after Prolonged Exercise: A Longitudinal Study during an Extreme Mountain Ultra-Marathon

**DOI:** 10.1371/journal.pone.0161855

**Published:** 2016-08-31

**Authors:** Pierre Andonian, Magalie Viallon, Caroline Le Goff, Charles de Bourguignon, Charline Tourel, Jérome Morel, Guido Giardini, Laurent Gergelé, Grégoire P. Millet, Pierre Croisille

**Affiliations:** 1 Department of Radiology, CHU Saint-Etienne, Université Jean-Monnet, Saint-Étienne, France; 2 Univ Lyon, UJM-Saint-Etienne, INSA, CNRS UMR 5520, INSERM U1206, CREATIS, F-42023, Saint-Etienne, France; 3 EuSpLM, Department of Clinical Chemistry, University of Liège, CHU Sart-Tilman, Liège, Belgium; 4 Department of Intensive Care, CHU Saint-Etienne, Saint-Etienne, France; 5 Department of Neurology and Neurophysiology, Mountain Medicine Center, Valle d’Aosta Regional Hospital, Aosta, Italy; 6 Institute of Sport Sciences of the University of Lausanne (ISSUL), Lausanne, Switzerland; Universite de Nantes, FRANCE

## Abstract

In sports medicine, there is increasing interest in quantifying the elastic properties of skeletal muscle, especially during extreme muscular stimulation, to improve our understanding of the impact of alterations in skeletal muscle stiffness on resulting pain or injuries, as well as the mechanisms underlying the relationships between these parameters. Our main objective was to determine whether real-time shear-wave elastography (SWE) can monitor changes in quadriceps muscle elasticity during an extreme mountain ultra-marathon, a powerful mechanical stress model. Our study involved 50 volunteers participating in an extreme mountain marathon (distance: 330 km, elevation: +24,000 m). Quantitative SWE velocity and shear modulus measurements were performed in most superficial quadriceps muscle heads at the following 4 time points: before the race, halfway through the race, upon finishing the race and after recovery (+48 h). Blood biomarker levels were also measured. A significant decrease in the quadriceps shear modulus was observed upon finishing the race (3.31±0.61 kPa) (p<0.001) compared to baseline (3.56±0.63 kPa), followed by a partial recovery +48 h after the race (3.45±0.6 kPa) (p = 0.002) across all muscle heads, as well as for each of the following three muscle heads: the rectus femoris (p = 0.003), the vastus medialis (p = 0.033) and the vastus lateralis (p = 0.001). Our study is the first to assess changes in muscle stiffness during prolonged extreme physical endurance exercises based on shear modulus measurements using non-invasive SWE. We concluded that decreases in stiffness, which may have resulted from quadriceps overuse in the setting of supra-physiological stress caused by the extreme distance and unique elevation of the race, may have been responsible for the development of inflammation and muscle swelling. SWE may hence represent a promising tool for monitoring physiologic or pathological variations in muscle stiffness and may be useful for diagnosing and monitoring muscle changes.

## Introduction

Measuring muscle mechanical properties in the setting of pathological states, such as neuromuscular diseases, has attracted great interest, as mechanical property measurements can be used to monitor neuromuscular disease courses or potential treatment-elicited improvements in neuromuscular diseases [1–4]. In sports medicine, there is increasing interest in quantifying the elastic properties of skeletal muscle, especially during extreme muscular solicitation, to improve our understanding of the impact of alterations in skeletal muscle stiffness on resulting pain or injuries in athletes, as well as the mechanisms underlying the relationships between these parameters [5].

Various techniques for estimating muscle tissue stiffness have been proposed, but they often require maximal voluntary contractions from subjects that are painful or physically demanding, which therefore limits their clinical applicability, accuracy and reproducibility [6,7]. Ultrasound imaging is a very promising alternative, as it provides muscle damage-related anatomical information and also enables the performance of quantitative wave velocity measurements in tissues, which have been shown to be related to muscle stiffness [8–11]. Shear-wave elastography (SWE) is an ultrasound-based technique that characterizes tissue elastic properties based on the propagation of remotely induced shear waves [12–14]. Unlike conventional ultrasound elastographic methods, SWE is not based on manual compression or the extent of tissue displacement [15]; thus, its data are more reliable, provided that an appropriate experimental setup is used. SWE calculates tissue stiffness based on tissue shear wave propagation velocity measurements. SWE is a non-invasive, quantitative, and real-time diagnostic imaging technique that is particularly well-adapted for in vivo investigations of skeletal muscle. When performed under well-controlled conditions that ensure reproducibility, SWE has been shown to be a reliable technique for investigating muscle biomechanical properties [16–20].

A few studies have explored the ability of SWE to evaluate the physiological responses of resting muscles (i.e., muscles that are not contracting or stretching during measurement) to short-term active or passive muscular solicitation [21,22] using eccentric injury models and plantar flexor [23,24] or elbow flexor eccentric contraction models [25]. Green et al. [23] explored the effects of downward running on a treadmill on the plantar flexor muscles under experimental conditions using magnetic resonance elastography (MRE). To our knowledge, no study has investigated elastic changes in human muscle injury models using a prolonged-racing model, particularly under extreme conditions characterized by prolonged loading. Therefore, exploring and validating the capability of a quantitative tool to assess changes in skeletal muscle properties during extreme solicitation is of great interest to us, as this quantitative tool may be useful for improving protective strategies and preventing injuries.

Mountain ultra-marathons (MUMs) have become increasingly popular within the last decade and are considered an outstanding model for investigating adaptive responses to extreme loads and stress [26]. MUMs induce eccentric quadriceps muscle contractions that impose large amounts of mechanical strain and ultimately lead to muscle damage [27,28], cytoskeletal alterations and neuromuscular functional impairments [28–30]. MUMs therefore represent a unique opportunity to assess the ability of SWE to evaluate changes in muscle stiffness, as well as to determine whether new quantitative indices derived from SWE measurements can be useful biomarkers for monitoring changes in muscle stiffness under extreme stress and whether these indices have the necessary sensitivity and accuracy for evaluating the effects of recovery on muscle stiffness.

The present study aims to evaluate the capability of SWE to track quadriceps muscle stiffness variations before, during and after the world's most challenging extreme mountain ultra-marathon.

## Materials and Methods

### Subjects and experimental study design

Subjects were recruited via mail and public announcements issued to registered runners by race organizers. Fifty experienced runners volunteered for and provided informed written consent to participate in this study. The experimental design of the study was approved by the local ethics committee of the Azienda Regionale Sanitaria USL della Valle d’Aosta, Direzione Meida di Presidio (acceptance n°900-18/08/2014), and all experiments were conducted in accordance with the Helsinki Declaration (2001). Demographic data regarding the study population are displayed in **Table 1**.

**Table 1:** Demographic and training profile data.

	Pre	Mid	Finish	Recovery
N	50	31	27	27
Sex (male/female)	46/4	31/1	30/1	29/0
Age (years)	43 ± 9.1	43 ± 8.6	43 ± 8.7	43 ± 8.6
Height (meters)	1.75 ± 6.2	1.75 ± 6.4	1.75 ± 6.3	1.75 ± 5.6
Weight (kg)	72.2 ± 8	71.7 ± 8.2	70.7 ± 7.2	70.8 ± 7.3
BMI (kg.m^-2^)	23.6 ± 2.0	23.4 ± 2.0	23.1 ± 2.1	23.1 ± 2.0
Body temperature (°C)	36.2 ± 0.9	37.3 ± 0.5	37.3 ± 0.5	37.1 ± 0.7
Pain	0.00 ± 0	4.1 ± 2.9	3.6 ± 2.9	1.08 ± 1.7
Training/week (n)	3.94 ± 1.7	3.94 ± 1.5	3.94 ±1.5	3.94 ± 1.5
Running experience (years)	14.2 ± 10.4	13.5 ± 10.5	13.3 ± 10.6	13.1 ± 9.8
Experience in ultra-marathons (years)	5.3 ± 3.6	5.5 ± 3.6	5.6 ± 3.6	5.3 ± 3.6
Previous ultra-marathons (n)	13 ± 10	11 ± 9	11 ± 9	11 ± 9
Limb dominance (R/L)	43/7	27/4	23/4	23/4

All values are presented as the mean (standard deviation)

Thigh pain was quantified on a visual analog scale

Body mass index (BMI) was calculated as weight/height squared (kg·m^-2^)

Pre, Mid, Finish and Recovery were the four key measurement time points:

Pre (pre-race) measurements were performed within 4 days before the race

Mid (mid-race) measurements were performed at the mid-point of the race (148.7 km, D+9270 m)

Finish measurements were performed at the end of the race, within 1 h after finishing

Recovery measurements were performed after 48–72 h of recovery

The Tor des Geants^®^ is a 330 km-long ultra-distance running trail in the Valley of Aosta (Italy) featuring considerable positive/negative elevation changes (+24,000 m). It is considered one of the most difficult (if not the most difficult) mountain marathon races in the world because it is an ultra-endurance activity characterized by high-altitude exposure and sleep deprivation. The altitude along the course ranges between 322 and 3.300 m, and the course features 25 mountain passes over 2000 m. The maximum time allowed to complete the race is 150 h. The best finishing time of 71 h 49 min was achieved in the 2014 race, which featured 740 starters and 446 (60%) finishers (http://www.tordesgeants.it/).

The present experiment was a longitudinal study in which assessments were repeated at the following four key time points: before the race, during the face, upon finishing the race and after the race. The first assessment (Pre) was performed within 4 days before the race. The second assessment (Mid) was performed at the mid-point of the race (148.7 km, D+9270 m). At the end of the race, each athlete was transported to the laboratory by car and evaluated for a third time (Finish) within 1 h after finishing the race. A final assessment (Recovery) was performed after 48–72 h of recovery. Body weight (kg) and body temperature (°C) measurements were recorded during each session. Prior to the initial session, questionnaires were administered to collect data regarding subject training experience.

### Blood biomarker collection and analysis

Blood samples were collected during each session within 10 minutes of arrival and prior to each ultrasound. Samples were drawn from an antecubital vein into a dried, heparinized or EDTA tube, depending on the analysis being performed. Both tubes were immediately centrifuged for 10 minutes (3500 RPM). Because it was not possible to carry out all analyses on the same day using point-of-care technology, plasma and serum samples were frozen at −80°C within 20 minutes of blood collection and stored for later analyses of muscle injury markers and biochemical parameters (**Table 1)**. Lactate was measured directly using an Accutrend Lactate Analyzer (Roche Diagnostics, Manheim, Germany). All hematology parameters (hemoglobin, red blood cells, white blood cells) were analyzed directly using a pocH-100i™ Automated Hematology Analyzer (Sysmex, Villepinte, France). Cobas 8000 and Cobas 6000 Modular Analyzers (Roche Diagnostics, Manheim, Germany) were used to perform serial CK, CKMB, hsTnT, NT-proBNP, MYO, and hsCRP measurements, as well as serial electrolyte, protein and hepatic and renal biomarker measurements.

### ShearWave^TM^ elastography (SWE) measurements: imaging protocols, procedures and analyses

An Aixplorer ultrasound system (version 8.0; Supersonic Imagine, Aix-en-Provence, France) equipped with ShearWave^TM^ Elastography and coupled with a linear transducer (4–15 MHz, SuperLinear 15–4; Vermon, Tours, France) was used in shear-wave elastography mode (musculoskeletal preset), as previously described in detail [12,31]. Briefly, this method is based on ultrafast ultrasound sequences that are performed to capture shear wave propagation. The shear-wave displacement field as a function of time is retrieved via one-dimensional cross-correlation of successive radio frequency signals along the ultrasound beam axis. The shear waves speed is then determined in each pixel of the resultant image using a time of flight algorithm on the displacement movies. Assuming linear elastic behavior [12,32] and a constant muscle mass density of 1000 kg·m^−3^, the shear modulus correlates directly with the shear-wave propagation velocity measurements.

**Fig 1:**
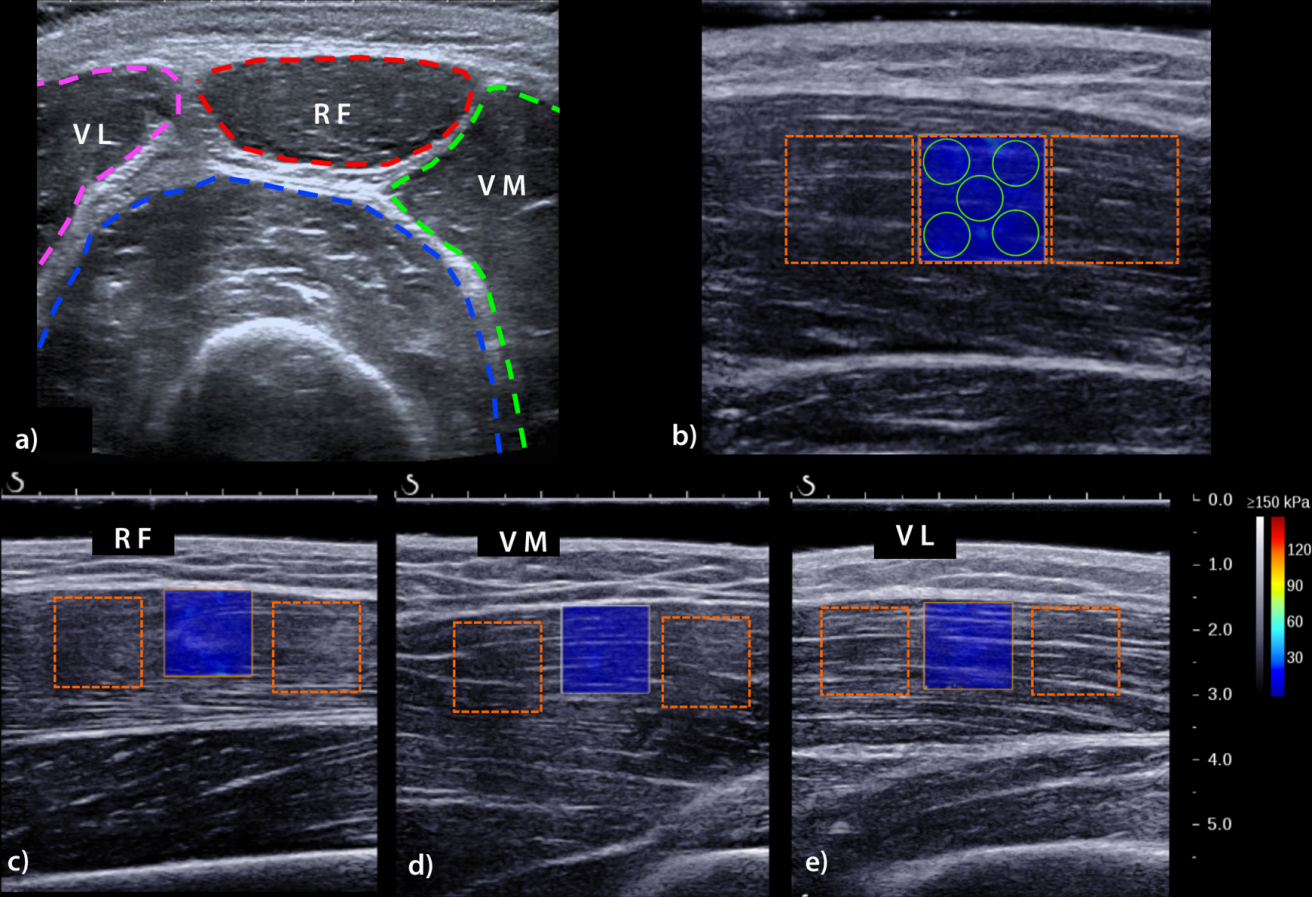
a) US image obtained in axial plane showing the different areas of interest within the quadriceps muscle: the RF, rectus femoris (red); the VL, vastus lateralis (pink); and the VM, vastus medialis (green). b) Elastographic data collection: 5 circular 5 mm-diameter ROIs (Q boxes) were manually placed within each squared SWE box by the same radiologist, who was experienced in performing musculoskeletal ultrasound. For example, this picture shows the positions of the Q boxes for the RF. The operator was blinded to the quantitative shear modulus data. c), d), E) and f) are in-plane fiber-aligned US images of the RF, VM, VL, respectively. The orange-dotted square ROIs correspond to the three sequential positions of the SWE-boxes used for the SWE measurements. Thus, at each investigation time, 9 SWE boxes were saved (3 for the RF, 3 for the VM and 3 for the VL). Note the absence of gas bubbles within the thick gel layer. The X- and Y-scales are, respectively, the in-plane and depth distances in centimeters.

To carefully standardize lower leg measurements and muscle positions across all sessions, joint angles were set such that the quadriceps were as slack as possible. All subjects were examined in the supine position with their knees passively flexed at 20°. Position was controlled with the same foam backing and contention at the feet level (see **Fig 1**).

Subjects were asked to stay as relaxed as possible. B-mode ultrasound was used to identify anatomical structures and to determine the optimal transducer locations for each muscle to maximize the alignment between the transducer and the directions of the muscle fibers. The following three superficial heads of the right quadriceps were sequentially studied: 1) the *rectus femoris* (RF), 2) the *vastus medialis* (VM) and 3) the *vastus lateralis* (VL).

Appropriate probe alignment was achieved when the transducer was parallel to the skin plane and when several thin fascicles could be traced across the image without interruption [33] (**Fig 1**D, **1**E and **1**F), as recommended by Gennisson et al., who found that shear waves propagate longitudinally along muscle fibers much more readily than they propagate perpendicularly or at any other rotation interval. This finding was confirmed by others, indicating that parallel transducer orientations provide the most reliable muscle elasticity measurements [16].

All measurements were performed by the same radiologist (4 years of experience). To maximize inter-day reliability and minimize the duration of transducer re-positioning at the same location during subsequent sessions in each subject, 4 permanent waterproof skin landmarks were drawn with a marker under 2D-mode monitoring during the Pre session. A first line, which extended across the thigh, was drawn 15 cm above the upper edge of the patella, perpendicular to the patella-ASIS (anterior superior iliac spine) axis. Three additional lines were drawn parallel to the longitudinal axis of the center of the RF, VM and VL and were aligned with the centers of each muscular belly head. Fibrous sagittal septa were avoided to maximize homogeneity within the SWE box. The abovementioned skin landmarks always remained at least partially visible but were always refreshed to ensure that they remained in place for subsequent sessions.

**Fig 2:**
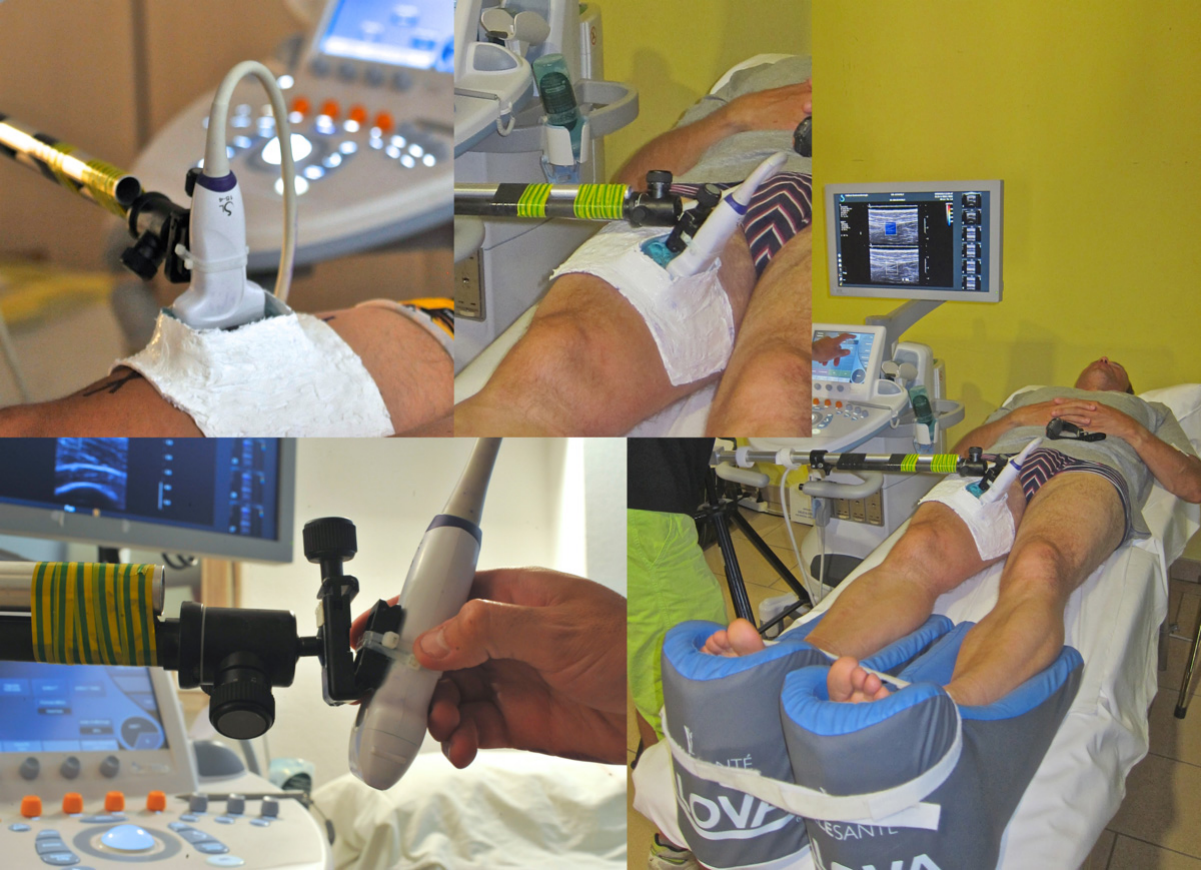
Measurement protocol and subject positioning. An articulated arm ensures that no contact occurs between the transducer and the thigh. Acoustic coupling is ensured using a home-designed silicon pool conforming to the shape of the leg that is filled with bubble-free acoustic gel. All subjects were placed in the supine position using a feet holder to ensure that the quadriceps femoris muscle remained at rest. To maximize inter-day reliability and hasten re-positioning, 4 indelible skin lines were traced during the Pre session (with a waterproof marker). The first line extended across the thigh and was drawn 15 cm above the upper edge of the patella, perpendicular to the patella-ASIS (anterior superior iliac spine) axis. The remaining three lines were drawn parallel to the longitudinal axis of the center of each of the three muscle heads (RF, VM, VL). Per the PLOS ONE policy regarding papers including identifying or potentially identifying, information, each subject was informed of the terms of the PLOS open-access (CC-BY) license and provided permission for the publication of these details under the terms of the license.

It is well known that soft tissue stiffness measurements are intrinsically modified if compression is produced by the transducer itself [17,34,35]. Thus, special care was taken to avoid inducing pressure on each muscle while preserving optimal probe coupling during each measurement. A custom-made system was developed to enable 1) precise and ergonomic transducer positioning with all required degrees of freedom for optimal positioning and 2) transducer position-locking once optimal locations and orientations were obtained. Then, to ensure that no pressure was exerted during measurement by the transducer itself while maintaining optimal acoustic coupling, a flexible silicon “pool” was designed. This device conformed to the shape of the anterior thigh but allowed the maintenance of a 2 cm-thick layer of ultrasound gel at the target location where the probe was partially immersed. A regular 5-mm ultrasound gel layer between the transducer and the skin minimized the application of pressure and allowed optimal acoustic coupling (**Fig 2**).

To remove all air bubbles from the gel (as air bubbles may represent potential ultrasound interfaces), the gel was pre-heated slightly, and any remaining bubbles within the silicon pool were subsequently aspirated with a 20 cc syringe. This precaution proved important, as many bubbles that were not visible at low altitudes were observed at Courmayeur (1300 m; Pre, Finish and Recovery sessions) and were attributed to decreases in barometric pressure.

Finally, after the anatomical locations and longitudinal fiber orientations of each superficial right quadriceps head were confirmed using 2D mode, the user was positioned such that a fixed-size square region of interest (ROI) (1.2 cm^2^) delimiting the elastographic field-of-view (SWE box), i.e., a region of interest where shear-wave propagation was analyzed within the muscle, was visible.

A short acquisition time delay is required to measure the mean shear modulus, irrespective of the size of the ROI or the muscle and tendon under study [17]. Therefore, a 5 s delay was consistently used before capturing each 2D map. Acquisition was performed when the real-time color map was as homogeneous as possible. Three successive SWE acquisitions were performed for each muscular head with the transducer in a fixed position to assess SWE reproducibility. Care was taken to avoid focal penetration defects or fibrous septa (**Fig 1**). The operator was blinded to the quantitative values obtained via SWE. The measurements were similar at each of the 4 investigation time points and were carried out as soon as possible after the cessation of physical effort (<15 min). During the Mid and Finish sessions, the SWE investigations began after an average delay of 15 minutes. The entire protocol lasted approximately 10 minutes.

Assuming linear elasticity [12,32], the muscle shear modulus was calculated as *μ* = *ρ*.*Vs*^2^, where *ρ* is the muscle mass density (1000 kg·m ^-3^), which is assumed to be constant, and *Vs* is the shear-wave velocity. Under these conditions, *μ* can be readily calculated from the shear-wave propagation velocity and tissue density [36] and is directly correlated with the shear wave propagation velocity.

The shear modulus (*μ*) and shear wave velocity (*Vs*) values were both voluntarily reported to allow comparisons with other studies reporting *Vs* or *μ* values.

DICOM images were transferred to an OsiriX workstation (Pixmeo, Geneva, Switzerland) for analysis using a dedicated analysis plugin (QBox, 1.0, Supersonic Imaging). This plugin allows direct extraction of elasticity metrics in circular ROIs within SWE color maps. To avoid artefacts in the circular ROIs, 5 ROIs (5 mm diameter) were placed within a given square SWE color map (**Fig 1B**). As 3 SWE acquisitions were performed in succession for each muscular head, 45 stiffness measurements were available at each measurement time point for each subject. Average muscular stiffness metrics (*μ* and *Vs*) were calculated for each quadriceps muscle and measurement time point to identify variations that could occur as a function of distance during the race and after 2 days of recovery.

### Statistical analysis

Data were initially screened for normality using the Kolmogorov-Smirnov tests, and all results pertaining to quantitative variables are reported as the mean ± standard deviation (SD).

Intra-session shear modulus measurement reliability was assessed by calculating the intraclass correlation coefficient (ICC) using a two-way random effect (consistency) model and standard error of measurements (SEMs) statistics. This approach quantifies the degree of agreement or reproducibility among all 15 measurements (5 ROIs in 3 slices) acquired consecutively during each session at each time point for the same subject by the same observer and without a posteriori exclusion of any data points according to image quality. The level of reliability was classified using ICCs defined as moderate (0.70–0.79), good (0.80–0.89)) or high (0.90) [37,38]. ICC values were compared using the Fischer Z test.

Changes in stiffness metrics (*μ* and *Vs*) among measurement sessions (Pre-race, Mid, Finish, and Recovery sessions) and muscle heads (RF, VM, VL) were assessed using a two-factors (session and muscle) fixed-effects repeated measures ANOVA model. The Box's conservation correction factor was applied to account for sphericity violations. Based on that model, post hoc pairwise comparisons were subsequently performed, followed by Bonferroni correction.

Pearson’s correlation coefficients were calculated to determine the relationships between stiffness parameters and biologic, demographic and training variables.

For all analyses, significance was accepted at p<0.05. Cohen’s *d* effect size [39] was reported where appropriate, and effect sizes of 0.2, 0.5 and 0.8 were classed as small, moderate and large, respectively. We also reported the common language effect size statistic (CL), which expresses the likelihood that a randomly sampled individual measurement from one group has a higher value than a randomly sampled measurement from another group [40], using Excel Spreadsheet version 15.24 for Mac (Microsoft Corporation par Impressa Systems, Santa Rosa, Californie), as recommended by Laken [41]. All other statistical analyses were performed using Stata 14 (College Station, TX) statistical software.

## Results

Demographic data are reported in **Table 1**. Of the 50 enrolled participants, 31 (66%) reached the mid- point and were examined (17 dropouts, 1 runner declined investigation). Twenty-seven finishers completed the race (54%) in an average time of 126±14 h.

### Measurement reliability

As shown in **Table 2**, the intra-session reliability of the shear modulus measurements for the Pre session was good for the VL and RF (ICC = 0.83 and 0.89) and high for the VM (ICC = 0.93), and no significant differences in reliability were noted among the quadriceps muscles (p = 0.12). Reliability remained within the same range during the Mid and Finish sessions, although the *μ* for the RF exhibited slightly higher reliability (ICC = 0.91) than the corresponding measurements for the *VM* and *VL* (ICC = 0.88) (p = 0.24). Intra-session reliability varied from good (ICC = 0.88 during the Pre and Mid sessions) to high (ICC = 0.90 and 0.92 at the Finish and Recovery sessions, respectively), and no significant differences in reliability were noted among the times (p = 0.16).

**Table 2:** Relative (intraclass coefficient) and absolute (standard error of measurement) shear modulus (*μ*) reliability.

Time	Muscle	ICC (95% CI)	SEM (kPa)
	RF	0.89 (0.84–0.93)	0.16
1(Pre)	VM	0.9 (0.89–0.96)	0.12
	VL	0.83 (0.75–0.89)	0.19
	global	0.90 (0.86–0.94)	0.20
	RF	0.92 (0.88–0.96)	0.15
2(Mid)	VM	0.86 (0.78–0.92)	0.18
	VL	0.87 (0.79–0.93)	0.19
	global	0.88 (0.82–0.94)	0.18
	RF	0.92 (0.87–0.95)	0.15
3(Arrival)	VM	0.85 (0.75–0.91)	0.16
	VL	0.93 (0.89–0.96)	0.13
	global	0.93 (0.89–0.96)	0.17
	RF	0.91 (0.85–0.95)	0.14
4(Recovery)	VM	0.93 (0.88–0.96)	0.14
	VL	0.92 (0.87–0.95)	0.14
	global	0.92 (0.87–0.96)	0.14

*RF*, *rectus femoris*; *VM*, *vastus medialis*; *VL*, *vastus lateralis; CI*, confidence interval; *SEM*, standard error of measurement used as an indicator of absolute reliability

### Shear modulus *(*μ) and shear wave velocity *(Vs)* changes among muscle heads and measurement times

At the Pre session, the overall shear modulus was 3.6±0.6 kPa, and the shear wave velocity was 1.88±0.17 m.s^-1^ when all measurements were pooled. μ was significantly lower in the VM (2.97±0.48 kPa; p<0.001) than in the RF (3.84±0.49 kPa) and VL (3.85±0.47 kPa). Furthermore, Cohen’s d effect size value (d = 1.83) suggested a high practical value. The CL effect size indicated that after controlling for individual differences, the likelihoods that a measure of *μ* scored lower in the VM than in the VL or RF were 89% and 90%, respectively.

Vs was also significantly lower in the VM (1.72±0.14 m.s^-1^) than in the RF (1.96±0.13 m.s^-1^) (p<0.001, d = 1.77, CL effect size = 89%) and VL (1.96±0.12 m.s^-1^) (p<0.001, d = 1.84, CL effect size = 90%).

The average muscular stiffness metrics (*μ* and *Vs*) for each quadriceps muscle and measurement time point are reported in **Table 3**.

**Table 3:** Longitudinal shear modulus (*μ*) and shear wave velocity (*Vs*) variations over time.

Muscle		Pre	Mid	Finish	Recovery
Rectus femoris	*μ* (kPa)	3.84 (0.49)	4.04 (0.71)	3.56 (0.57)	3.8 (0.47)
	*Vs* (m.s^-1^)	1.96 (0.13)	2.00 (0.18)	1.88 (0.16)	1.95 (0.12)
Vastus medialis	*μ* (kPa)	2.97 (0.48)	2.96 (0.49)	2.81 (0.45)	2.95 (0.48)
	*Vs* (m.s^-1^)	1.72 (0.14)	1.72 (0.14)	1.67 (0.14)	1.71 (0.14)
Vastus lateralis	*μ* (kPa)	3.85 (0.47)	3.87 (0.53)	3.56 (0.48)	3.48 (0.5)
	*Vs* (m.s^-1^)	1.96 (0.12)	1.96 (0.13)	1.89 (0.13)	1.89 (0.13)
**Without muscle head distinction**	*μ* **(kPa)**	**3.56** (**0.63)**	**3.62** (**0.75)**	**3.31** (**0.61) ‡**	**3.45** (**0.6- ‡**
	***Vs*** (m.s^-1^)	**1.88** (**0.17)**	**1.89** (**0.20)**	**1.81** (**0.17)**	**1.85** (**0.16)**

Values are presented as the mean (SD) of the shear modulus (*μ*) and shear wave velocity (*Vs*)

Pre, Mid, Finish and Recovery correspond to each measurement time

ANOVA revealed that the 2 main factors exerted significant effects, as there were significant differences among the muscle heads (F(2,336) = 103.5, p<0.001) and measurement sessions F(3,336) = 4.63, p = 0.003), but there were no interaction between the muscle heads and changes over time F(6,336) = 0.897, p<0.49). The *vastus medialis* μ and *Vs* values remained lower than the corresponding RF and VL values at the Mid (p<0.001, d(*μ*) = 1.75, d(*Vs*) = 1.71) and Finish sessions (p<0.001, d(*μ*) = 1.47, d(*Vs*) = 1.31) and at the Recovery session (p<0.01, d(*μ*) = 1.78, d(*Vs*) = 1.33).

**Fig 3:**
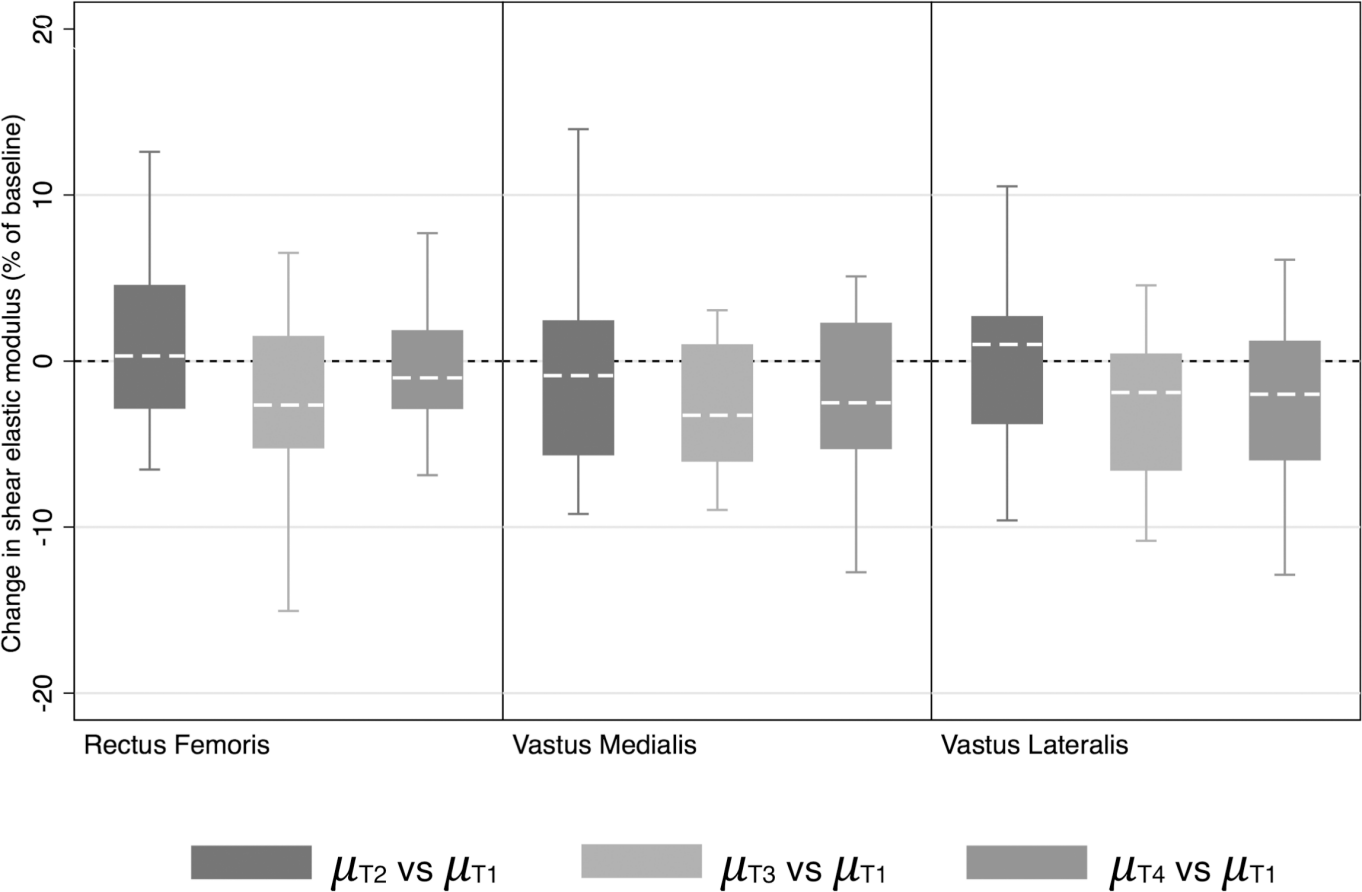
Change in muscle head stiffness (shear modulus). Changes observed in the three muscle heads (RF, VM and VL) at the three measurement times (Mid, Finish, and Recovery) are expressed as percentages of their baseline values, or their Pre values. The error bars denote the 95% confidence interval, and the boxes denote the 25^th^–75^th^ percentiles with the median. The asterisks indicate that the changes from baseline are significant based on the statistical analysis of the raw data, which is presented in the Results section.

While there was no difference in *μ* between the Pre- and Mid sessions (p = 0.81), there was a significant decrease in *μ* at the Finish session (p<0.001)—with a moderate effect size (d = 0.56)—which persisted at the Recovery (p = 0.002) session, with a small-to-moderate effect size (d = 0.35), compared to the Pre session. The CL effect size indicates that after controlling for individual differences among the time points, the likelihoods that *μ* would be decreased were 71% and 63% at the Finish and Recovery sessions, respectively. **Fig 3** shows the percent changes (from baseline) in the shear modulus for each individualized muscle and displays similar trends with respect to the percent changes in the shear modulus of each muscle.

### Relationships between stiffness properties and changes in clinical and biological covariates

At baseline, there was a significant negative relationship between both *μ* and *Vs* and BMI (r = -0.20, p = 0.01), as well as a weak negative relationship between these parameters and age (r = -0.14, p = 0.074); however, there were no relationships between these parameters and leg dominance (r = -0.06, p = 0.52), training or trail experience (i.e., the total number of ultra-endurance trails completed) (p = 0.20), numbers of years participating in ultra-trail races (p = 0.62), numbers of years participating in running (p = 0.71), or training volume (p = 0.52).

Among finishers, there were negative relationships between absolute *μ* and *Vs* and leg pain during the race (*μ*: r = -0.28, p = 0.01; *Vs*: r = -0.22, p = 0.04), but there were no relationships between these parameters and performance (i.e., race duration, h) (*μ*: r = -0.08, p = 0.49; *Vs*: r = -0.08, p = 0.46).

The longitudinal variations in blood biomarkers throughout the race are shown in **Table 4**. Briefly, serum CK and myoglobin levels peaked at +7686% and +4889% at Mid, while serum LDH levels peaked at +268% at Finish and were elevated at Mid at +203% (all p<0.001). Total serum protein, creatinine, uric acid, WBC and neutrophil levels also increased significantly (p<0.001) and peaked at Mid. The percent change in *μ* between Pre and Finish was negatively correlated with the corresponding percent changes in creatinine (p = 0.01), GFR (p = 0.01), plasma osmolality (p = 0.02), and WBCs/neutrophils (p = 0.01), but not with the percent changes in other biomarkers (**Table 5**).

**Table 4:** Longitudinal variations in blood biomarkers throughout the ultra-marathon race.

	Pre	Mid	Finish	Recovery
**Creatine kinase** (UI.L^-1^)	133.29 (71.80)	**10379.69** (10135.55)**	4123.30 (3846.10)**	733.52 (626.31)**
**Myoglobin** (μg.L-^1^)	27.84 (8.55)	**1388.99** (1480.48)**	547.28 (436.49)**	86.78 (44.32)**
**LDH** (UI.L^-1^)	166.04 (29.87)	507.56 (255.70)**	**611.09** (258.48)**	428.23 (177.49)**
**Lactate** (mmol.L^-1^)	1.83 (0.40)	1.74 (0.52)	**2.28** (0.89)*	2.02 (0.54)
**C-reactive protein** (mg.L^-1^)	1.17 (1.78)	**20.45** (13.66)**	16.65 (15.0)**	5.87 (4.71)**
**Serum uric acid** (mg.dL-1)	36.11 (7.95)	**74.83** (22.11)**	56.09 (15.80)**	39.18 (9.38)
**Serum creatinine** (mg.dL^-1^)	0.97 (0.11)	**1.18** (0.18)**	1.13 (0.16)**	0.95 (0.10)
**GFR** (mg.L^-1^)	93.53 (11.67)	86.67 (16.21)*	91.85 (13.91)	**102.14** (8.54)**
**Sodium** (mmol.L^-1^)	140.62 (1.80)	155.92 (6.34)**	**160.49** (10.25)**	151.76 (10.19)**
**Potassium** (mmol.L^-1^)	4.06 (0.39)	4.06 (0.42)	3.98 (0.36)	4.24 (0.51)
**Serum total protein** (g.L^-1^)	72.63 (3.56)	**75.23** (4.25)**	74.57 (4.61)*	71.86 (5.42)
**Plasma osmolality** (mosm.L^-1^)	285.27 (3.68)	**290.70** (4.60)**	286.40 (6.35)	287.42 (4.86)*
**Hematocrit** (%)	43.53 (2.47)	**39.22** (2.82)**	37.92 (3.02)**	40.54 (2.84)**
**White blood cells** (x10³.μL^-1^)	7.02 (1.55)	**10.72** (2.13)**	8.51 (1.76)**	6.32 (1.46)
**Neutrophils** (x10³.μL^-1^)	4.17 (1.14)	**7.66** (2.18)**	5.93 (1.57)**	4.10 (1.24)

*LDH*, Lactate dehydrogenase

Glomerular filtration rate (GFR) was calculated according to the CKD-Epi formula

Bold values are peak changes over time

Values are shown as the mean (SD)

* p<0.05

** p<0.001 for post hoc paired comparisons with Pre values

**Table 5:** Percent changes in biomarker levels and shear modulus values between the Finish and Pre sessions (% change Finish/Pre).

	% change Finish/Pre	r	p
Creatine kinase (UI.L^-1^)	2697.7 (348.5	0.07	0.49
Myoglobin (μg.L-1)	2024.7 (168.1	-0.01	0.89
LDH (UI.L^-1^)	280.5 (16.4	0.08	0.46
Lactate (mmol.L^-1^)	21.2 (4.3	-0.12	0.28
C-reactive protein (mg.L^-1^)	2443.8 (226.1	-0.18	0.09
Serum uric acid (mg.dL-1)	63.1 (4.2	-0.09	0.41
Serum creatinine (mg.dL^-1^)	16.3 (2.5	-0.26	0.01
GFR (mg.L^-1^)	-0.26 (1.2	0.27	0.01
Sodium (mmol.L^-1^)	14.1 (0.6	-0.12	0.26
Potassium (mmol.L^-1^)	-0.8 (1.1	0.10	0.33
Serum total protein (g.L^-1^)	2.7 (0.5	-0.07	0.49
Plasma osmolality (mosm.L^-1^)	0.4 (0.2	-0.23	0.02
Hematocrit (%)	-12.4 (0.4	0.06	0.57
White blood cells (x10³.μL^-1^)	23.6 (2.8	-0.28	0.01
Neutrophils (x10³.μL^-1^)	51.8 (4.7	-0.26	0.01

*LDH*, Lactate dehydrogenase

Glomerular filtration rate (GFR) was calculated according to the CKD-Epi formula

Values are shown as the mean (SD)

P was calculated via post hoc paired comparisons between the Finish and Pre sessions

## Discussion

To our knowledge, this is the first study to provide evidence that non-invasive SWE has the capacity to monitor changes in muscle stiffness in a longitudinal human model of extreme prolonged mechanical stress.

We demonstrated that prolonged and mainly eccentric low-intensity exercises induce changes in quadriceps muscle stiffness that can be quantified by SWE. We observed significant decreases in *Vs* and *μ* between the Pre and Finish sessions (p<0.001) and milder but significant differences in these parameters between baseline and after more than 45 hours of recovery (p = 0.002), indicating that neither *Vs* nor μ returned completely to baseline. These global trends were observed across all muscle heads but were also detected when each muscle head was analyzed individually, as shown in **Table 3**. The three superficial muscle heads exhibited the same behavior at each of the 4 measurement times and exhibited similar changes in *μ*. Note that the VI muscle, the most solicited muscle with respect to eccentric contraction elicited during downhill running [42], was not assessed because of its depth, which made performing elastographic measurements via ultrasound difficult. It has been established that the magnitudes of the micro-lesions incurred during eccentric solicitation induced by MUMs are dependent on the muscle type, the level of force generated during the period and/or the amplitude of stretching, as well as the duration of exercise [27]. Consequently, we hypothesized that the VM, VL and RF muscles would exhibit similar behavior with respect to muscle fatigue and muscle solicitation during MUMs.

This study is one of the few to estimate *Vs* and *μ* on a non-contracted muscle and is the only one to estimate muscular stiffness at various stages (before, during, immediately after and 2 days after exercise) during a prolonged and extreme endurance solicitation, such as the Tor des Géants^®^ MUM. As comparisons with similar prolonged exercise models were not possible, we elected to first compare baseline values with values obtained after bouts of eccentric exercise. Regarding the concordance of our *μ* values with existing published data, our mean shear modulus value and standard deviation for the *rectus femoris* at the Pre session of 3.84±0.5 kPa were concordant with values of 4.3±1.2 kPa [17] and 3.2±0.4 kPa [18], which were obtained in previous studies. Regarding the VM, our mean resting shear modulus value was 3±0.5 kPa, which is lower than the following value reported by Botanlioglu et al.: 4.9±1.8 kPa [43]. Finally, regarding the VL, our mean shear modulus value was 3.9±0.5 kPa, while the values obtained by two of the abovementioned studies were 5.4±1.2 kPa [43] and 3.3±0.4 kPa [18].

Some of the differences between our resting values and those observed by Botanlioglu et al. [43] may be related to methodological differences between our acquisition protocol and theirs, as their study did not report any specific strategies for avoiding biases introduced by uncontrolled transducer pressure or uncertainty regarding measurement reproducibility. In our study, the Q box was placed during data acquisition, according to a rigorous protocol, and data processing was carried out separately by an operator who was blinded to the quantitative shear modulus data, which allowed us to avoid subjectivity bias. Under these measurement conditions, shear-wave imaging reproducibility (the quadriceps muscle without contraction) was excellent, as shown by the intra-session correlation measurements, which featured intra-session standard deviations that were among the lowest in the literature [17,43]. It is also worth noting that resting muscle measurements are easier to standardize than contracted muscle measurements and do not require any effort from the athlete.

The decreases in muscular stiffness observed in this study are also concordant with those observed by Giovanelli et al. and Garcia-Manso et al., who demonstrated decreases in *vastus lateralis* stiffness immediately after an uphill marathon [44] and decreases in biceps *femoris* stiffness 15 minutes after an Ironman triathlon [14], respectively, using invasive techniques, such as tension-myography (TMG) and muscle belly deformation. In sports with a high stretch-shortening cycle component (e.g., mountain running), repeated eccentric contractions (e.g., in the knee extensor, quadriceps or plantar flexor muscles) have considerable deleterious effects on muscular function (e.g., decreases in maximal voluntary contraction and alterations in excitation-contraction coupling) [45–49]. However, compared to runners participating in MUMs of different durations, including MUMs of shorter durations (e.g., UTMB, 20–46 h), runners participating in the Tor des Geants exhibited less inflammation and less muscle damage, probably as a result of lower concentric/eccentric contraction intensities due to lower velocities [50].

Most previous studies exploring changes in muscles stiffness after exercise used human muscle injury models featuring acute eccentric exercises characterized by increases in muscle stiffness resulting from increases in passive tension, as determined via clinical measurements that were often obtained using ergometers rather than SWE [51,52]. These measurements cannot isolate the stiffness of individual muscles and thus provide only global information regarding the behavior of several structures (e.g., muscles, tendons, nerves, and skin) acting around a given joint.

Regarding the studies that assessed muscle shear modulus changes after eccentric exercise, all reported changes after short exercise durations (≤30 minutes), but none explored muscle stiffness changes after extremely prolonged physical exercise comparable to that reported here (distance: 330 km, elevation: +24,000 m, duration: 126±14 h). Green et al. [53] reported modest increases in the shear modulus of the gastrocnemius medialis (GM) after 15 minutes of backwards walking (2 km/h) on an inclined treadmill, which they measured using a different technique (magnetic resonance elastography, not SWE). Guilhem et al. [24] reported a 28% increase in the GM shear modulus immediately after 10 sets of 30 maximal eccentric contractions of the plantar flexor muscles. Lacourpaille et al. [25] showed increases in the shear modulus of the elbow extensor muscles (in the stretched position) at 1 h and 48 h after three sets of ten maximal eccentric contractions, but these increases were significant only when the abovementioned muscles were in the stretched position. In the present study, muscles were analyzed in the slack position. As discussed by Warren et al. [54], comparisons of the results of various injury models may reveal the presence of significant heterogeneity, especially when comparing the results of experimental models (featuring acute eccentric contractions involving a single muscle group) with those of whole-body exercises performed under real conditions (featuring eccentric contractions performed during prolonged-racing models). Even models featuring acute eccentric contractions have been able to show differences in injury susceptibility and responsiveness between the elbow flexors and knee flexors, as the former appear to be more prone to suffering eccentric injuries without a clear explanation [54]. Therefore, in the event that the decreases in the shear modulus reported in the present study initially seem to contradict the results of previous studies, it is important to remember that not only did the measurements performed in this study involve different muscle groups, but they were also performed at time points and under muscle loading conditions that may be too different from those of other studies to authorize direct results comparisons: indeed, skeletal muscle responses to ultra-endurance testing are highly specific and are dependent on exercise intensity and duration [55]. Measurements were performed only in the slack position, as this position was considered the most achievable and reproducible position. Moreover, it was applicable at all measurement time points. This may be considered a limitation of the present study. It is worth noting that increases in muscle stiffness post-eccentric exercise have been attributed to perturbations of calcium homeostasis. As muscle fiber sensitivity to Ca2+ reportedly increases as muscles elongate [56], it is plausible that our results would have been different had we performed our measurements in a more stretched position.

Sonoelastography studies on non-contracted muscles have reported that the persistence of muscle stiffness changes may be exceptionally brief (with a return to the baseline within few minutes [21,57]) or less than 48 h [24,25].

**Fig 4:**
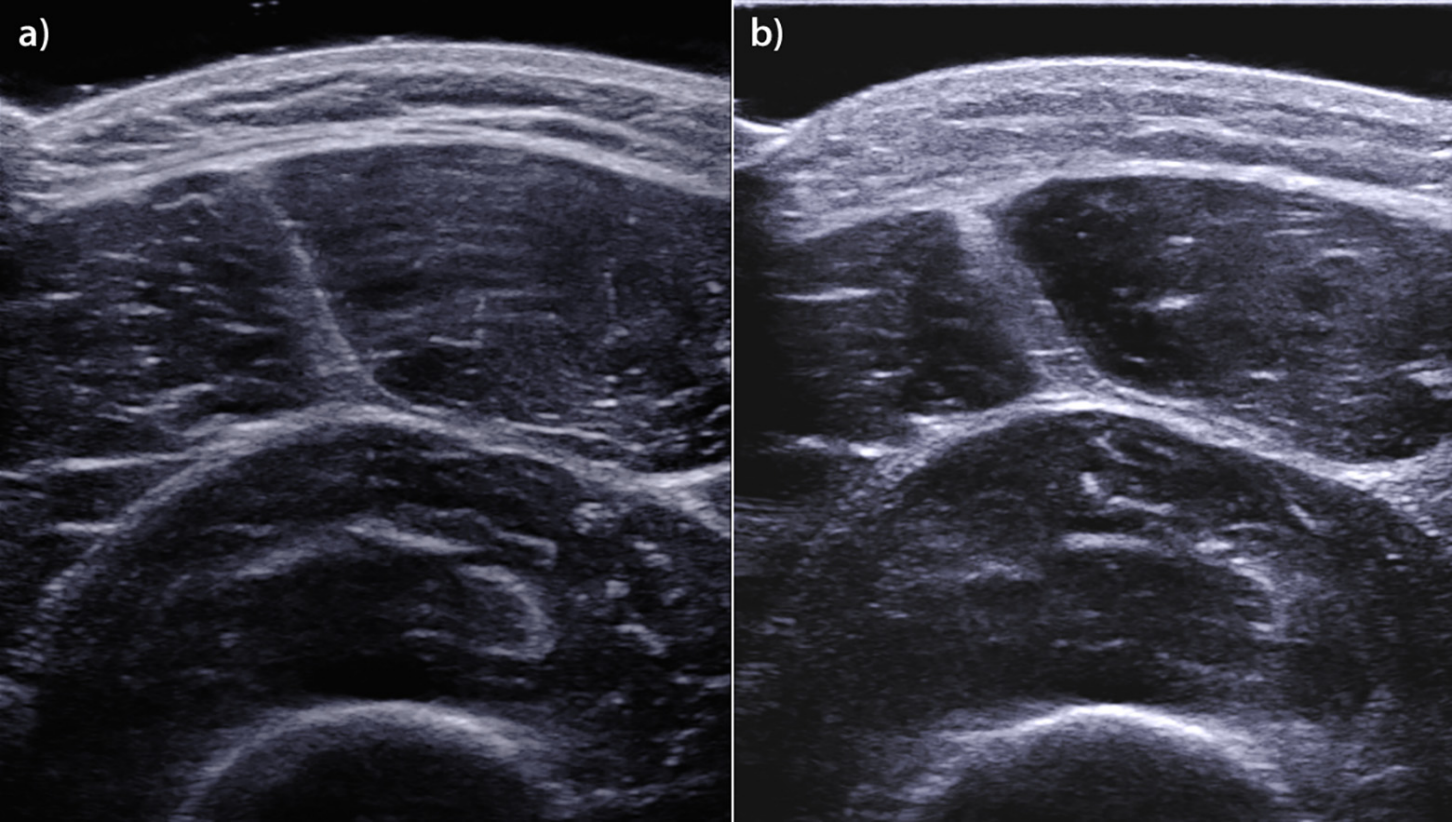
Edematous and architectural thigh muscle changes at the Finish assessment. US images of the quadriceps femoris muscle, which were obtained in the axial plane during the Pre session (a) and the Finish session (b). Note the edematous thickening of the skin and the muscle fascia in the image obtained during the Finish session.

The results of the present study indicate that our shear-wave velocity and shear modulus values obtained at 2 days after the Finish session remained different from the same values obtained at baseline. These results may be due to the specific load conditions of the Tor des Géants^®^ MUM. Increases in total body water, as well as the development of peripheral and muscular edema, have been reported in the context of ultra-marathon running. One study reported a 6% increase in total body water after a 1200 km run over 17 consecutive days [58], and another study reported an increase in total body water associated with tissue edema [59]. Such changes may be related to skeletal muscle inflammation. This hypothesis is supported by our B-mode US anatomical images, which clearly showed increases in fascial thickness and subcutaneous edema (**Fig 4**). These changes may be explained by extracellular water volume expansion caused by local inflammation. The large MUM-induced increase in total water content is interesting, as the shear modulus can be influenced by tissue mass density. It is therefore possible that the increases in muscle water counterbalanced the increases in muscle stiffness resulting from muscle damage.

Repetitive eccentric muscle contractions during MUMs result in microscopic muscle damage [27,60,61] and increased inflammation [50,62]. The authors also hypothesized that water accumulation contributes to tissue regeneration after muscle damage. However, Lacourpaille et al. noted an increase in the shear modulus that persisted in the presence of edema, as measured via T2-weighted MRI at 48 h after exercise [25]. Therefore, inflammation and swelling cannot be the main factors responsible for changes in elasticity, as suggested by Whitehead et al. [52]. Extracellular matrix (ECM) changes may play a role in muscle stiffness changes, as there is an established relationship between ECM changes and passive stiffness [63]. The rate of collagen turnover increases with exercise in a time-dependent manner following increases in myofibrillar protein synthesis, even during acute exercise [64]. Additionally, ultra-endurance exercise triggers autophagy-related and autophagy-regulatory gene expression, as shown by Jamart et al., who performed VL muscle biopsies following a 200 km ultra-marathon [55]. These changes are considered a response to extreme stress induced by the combination of energy deprivation and oxidative stress, which triggers the unfolded protein response [65,66]. As noted by Bell et al. [67], ultra-endurance exercises represent skeletal muscle stress-inducers, as they are characterized by increased energy demands triggering proteolysis and dysfunctional protein/organelle destruction.

As expected, we noted large increases in the levels of skeletal myocyte injury-related biomarkers (CK, myoglobin and LDH), which have been shown to reflect the extent of cellular damage [68,69]. Blood biomarkers remain indirect indices of muscle damage that are not consistently correlated with the extent of muscle damage. Our peak CK and myoglobin levels of 10,780 UI.L^-1^ and 1389 μg.L^-1^, respectively, were similar to but lower than those observed in a 166 km MUM (with CK and myoglobin levels of 13,500 UI.L^-1^ and 1730 μg.L^-1^, respectively) [70]. Interestingly, in our study, mean CK and myoglobin values peaked at the Mid-session (after 149 km) and decreased during the second half of the race. This biphasic response is likely related to decreases in runner speed during the second half of the race, Maufrais et al. demonstrated that runner speed is 32% slower during the second half of the Tor des Geants than the in first half [71]. Increases in uric acid levels can be explained by enhancements in protein catabolism, which occur during long-term exercise due to glycogen depot depletion [72], Increases in creatinine are related to increases in the catabolic metabolites of muscle damage [73]. Despite minor GFR fluctuations, the Mid (1.18 mg.dL^-1^) and Finish assessment creatinine values noted in this study (1.13 mg.dL^-1^) were higher than those noted by Millet et al. after the aforementioned 166 km race (1.03 dL^-1^). WBC activation, particularly neutrophil activation, triggers cellular and humoral inflammatory responses [74] characterized by elevated CRP levels and the presence of leucocytes in the extracellular and the extravascular spaces in response to long-term exercise [69,75]. Muscle damage inter-subject variability is related to many factors, including gender [76], age [77,78], and training status [79]. In our athlete population, there was no relationship between stiffness changes and the covariates reflecting trail-running experience or training level. We can also reasonably hypothesize that our group was a biased athlete population that could not be compared to those of previous studies, which compared the responses of trained subjects to those of untrained subjects [79]. Stiffness changes were significantly correlated with BMI, a finding consistent with those of recent works [80]. The weak relationship with age noted in this study is consistent with the results obtained by Manfredi et al., who noted that the VL muscle is more susceptible to damage after acute exercise in older patients than in younger patients [77]; however, Roth et al. noted no ultrastructural changes in this muscle or differences in its susceptibility to damage in older male subjects compared to younger male subjects [78]. We could not explore gender as a covariate because, as shown in our demographic data, most of the female athletes who participated in our study did not finish the MUM.

Early signs of cellular inflammation, such as increases in white blood cell counts and CRP levels, precede water redistribution from the intracellular to the extracellular compartment, leading to water accumulation in the extracellular compartment, i.e., edema. Therefore, decreases in *μ* may be associated with increases in edema, which occur more slowly than changes in blood biomarker levels. This may explain the absence of correlations between blood biomarker levels and *μ* at the Mid and Finish assessments, as blood biomarker levels may have changed more rapidly than *μ*, which decreased slightly later in conjunction with changes in edema.

The pro-inflammatory response has been shown to be triggered at the cerebral level via limb skeletal muscle sensory innervation pathways initiated by metabosensitive and mechanosensitive III/IV afferent neurons, which facilitate compensatory and/or protective delayed neural adjustments [81]. Therefore, we cannot rule out the possibility that inflammation induces changes in elasticity through neural inhibitory mechanisms in conjunction with decreases in muscle spindle sensitivity [82].

It is worth noting that lower quadriceps shear modulus values were obtained during a subsequent submaximal isometric torque-matched task [83], as shear modulus values are an indicator of lower force production caused by quadriceps muscle fatigue. However, neuromuscular fatigue does not necessarily correlate with running distance, as it was less prominent among the runners who completed the Tor des Géants than those who completed shorter MUMs [50]. Our study noted a decrease in *μ* during the MUM, which was probably related to inflammation and to the aforementioned decreases in force production, a finding consistent with that reported by Vitiello et al. [84]. Overall, the decreases in quadriceps stiffness noted in this study may be a consequence of the combination of specific MUM-induced changes (eccentric component, inflammatory responses and edema), which may represent a (relative) protective mechanism designed to combat the extreme load associated with the MUM.

Furthermore, in the present study, the decrease in VL stiffness was less pronounced than the decreases in VM and RF stiffness. Voloshina et al. demonstrated that when running on uneven terrain (such as during the Tor des Géants^®^), runners exhibit significant increases in RF and VM activity (electromyographic activity) compared with running on even ground, but no significant changes in VL activity [85,86], suggesting that the VM and RF are solicited more than the VL during prolonged MUMs, which may support the hypothesis that decreases in muscular stiffness are caused by specific prolonged muscular hyper-solicitation.

Because prolonged exercise leads to central and peripheral fatigue, it may be interesting to investigate the relationship among shear modulus changes, extracellular water increases and fatigue development.

In conclusion, using a model of prolonged and extreme mechanical stress, we showed that SWE monitored and highlighted changes in muscle stiffness and enabled us to gain a better understanding of muscle mechanics. We demonstrated that despite the accumulation of eccentric contractions induced by prolonged low-to-moderate intensity exercise associated with a 330 km race held at an altitude of +24,000 m, ultra-long running exercise leads to only moderate muscle damage and inflammation, confirming previous results indicating that long MUMs paradoxically induce less muscle damage [9].

The objective of our study was to investigate whether localized muscle stiffness quantification constitutes a non-invasive surrogate measurement of muscle damage that can be used in injury prevention programs or rehabilitation programs designed for athletes [9]. Unfortunately, the present study could not directly determine the extent of muscle damage and therefore could not correlate shear modulus changes with changes in damage indices. In addition, our results differed from those of several controlled models of damaging exercise; thus, further research is required before SWE can ultimately be used to quantify the effects of neuromuscular disorders on muscle function or the improvements in muscle function elicited by various treatments as a means of assessing the efficacy of these treatments, especially in the setting of inflammation.

Athletes and physicians were motivated to participate in this study to facilitate and encourage technological developments that may enhance our understanding of muscle physiology during extreme exercise and improve muscular lesion diagnoses, follow-up evaluations and care. The imaging community is interested in quantifying muscle properties using widely available and non-invasive methods to improve the diagnosis and monitoring of muscle injuries in athletes and patients [10,87].

Given that US enables imaging of muscle injuries and visualization of trauma signs on anatomical images, as well as shear modulus measurements and glycogen content quantification [88,89], it may be a useful and non-invasive means of investigating the relationship among force loss, muscle stiffness and performance in athletes.

## References

[1] Féasson L, Camdessanché JP, Mandhi El L, Calmels P, Millet GY. Fatigue and neuromuscular diseases. Annales De Réadaptation Et De Médecine Physique: Revue Scientifique De La Société Française De Rééducation Fonctionnelle De Réadaptation Et De Médecine Physique. 2006;49: 289–300, 375–384 10.1016/j.annrmp.2006.04.015 16780988

[2] Dietz V, Quintern J, Berger W. Electrophysiological studies of gait in spasticity and rigidity. Evidence that altered mechanical properties of muscle contribute to hypertonia. Brain: A Journal of Neurology. 1981;104: 431–449. 7272709 10.1093/brain/104.3.431

[3] Lehmann JF, Price R, deLateur BJ, Hinderer S, Traynor C. Spasticity: quantitative measurements as a basis for assessing effectiveness of therapeutic intervention. Archives of Physical Medicine and Rehabilitation. 1989;70: 6–15. 2916921

[4] Lieber RL, Steinman S, Barash IA, Chambers H. Structural and functional changes in spastic skeletal muscle. Muscle \& Nerve. Wiley Subscription Services, Inc., A Wiley Company; 2004;29: 615–627. 10.1002/mus.20059 15116365

[5] Mueller SM, Knechtle P, Knechtle B, Toigo M. An Ironman triathlon reduces neuromuscular performance due to impaired force transmission and reduced leg stiffness. Eur J Appl Physiol. 2015;115: 795–802. 10.1007/s00421-014-3051-2 25471270

[6] Leonard CT, Deshner WP, Romo JW, Suoja ES, Fehrer SC, Mikhailenok EL. Myotonometer Intra- and Interrater Reliabilities. Archives of Physical Medicine and Rehabilitation. 2003;84: 928–932. 10.1016/S0003-9993(03)00006-6 12808553

[7] Cowan SM, Bennell KL, Hodges PW. The test–retest reliability of the onset of concentric and eccentric vastus medialis obliquus and vastus lateralis electromyographic activity in a stair stepping task. Physical Therapy in Sport. 2000;1: 129–136. 10.1054/ptsp.2000.0036

[8] Brandenburg JE, Eby SF, Song P, Zhao H, Brault JS, Chen S, et al Ultrasound Elastography: The New Frontier in Direct Measurement of Muscle Stiffness. Archives of Physical Medicine and Rehabilitation. 2014;95: 2207–2219. 10.1016/j.apmr.2014.07.007 25064780 PMC4254343

[9] Hug F, Tucker K, Gennisson JL, Tanter M, Nordez A. Elastography for Muscle Biomechanics: Toward the Estimation of Individual Muscle Force. Exercise and Sport Sciences Reviews. 2015;43: 125–133. 10.1249/JES.0000000000000049 25906424

[10] Lacourpaille L, Hug F, Guével A, Péréon Y, Magot A, Hogrel J-Y, et al Non-invasive assessment of muscle stiffness in patients with duchenne muscular dystrophy: Short Report. Muscle \& Nerve. 2015;51: 284–286. 10.1002/mus.24445 25187068

[11] Eby SF, Cloud BA, Brandenburg JE, Giambini H, Song P, Chen S, et al Shear wave elastography of passive skeletal muscle stiffness: Influences of sex and age throughout adulthood. Clinical Biomechanics. 2015;30: 22–27. 10.1016/j.clinbiomech.2014.11.011 25483294 PMC4298479

[12] Bercoff J, Tanter M, Fink M. Supersonic shear imaging: a new technique for soft tissue elasticity mapping. IEEE Trans Ultrason, Ferroelect, Freq Contr. 2004;51: 396–409. 10.1109/TUFFC.2004.1295425 15139541

[13] Shigao C, Urban M, Pislaru C, Kinnick R, Yi Z, Aiping Y, et al Shearwave dispersion ultrasound vibrometry (SDUV) for measuring tissue elasticity and viscosity. IEEE Trans Ultrason, Ferroelect, Freq Contr. 2009;56: 55–62. 10.1109/TUFFC.2009.1005 PMC2658640 19213632

[14] Palmeri ML, Wang MH, Dahl JJ, Frinkley KD, Nightingale KR. Quantifying hepatic shear modulus in vivo using acoustic radiation force. Ultrasound in Medicine \& Biology. 2008;34: 546–558. 18222031 10.1016/j.ultrasmedbio.2007.10.009 PMC2362504

[15] García-manso JM, Rodríguez-Ruiz D, Rodríguez-Matoso D, de Saa Y, Sarmiento S, Quiroga M. Assessment of muscle fatigue after an ultra-endurance triathlon using tensiomyography (TMG). Journal of Sports Sciences. 2011;29: 619–625. 10.1080/02640414.2010.548822 21391085

[16] Gennisson J-L, Deffieux T, Macé E, Montaldo G, Fink M, Tanter M. Viscoelastic and Anisotropic Mechanical Properties of in vivo Muscle Tissue Assessed by Supersonic Shear Imaging. Ultrasound in Medicine \& Biology. 2010;36: 789–801. 10.1016/j.ultrasmedbio.2010.02.013 20420970

[17] Kot BCW, Zhang ZJ, Lee AWC, Leung VYF, Fu SN. Elastic modulus of muscle and tendon with shear wave ultrasound elastography: variations with different technical settings. PLoS ONE. Public Library of Science; 2012;7: e44348 10.1371/journal.pone.0044348 22952961 PMC3432131

[18] Lacourpaille L, Hug F, Bouillard K, Hogrel J-Y, Nordez A. Supersonic shear imaging provides a reliable measurement of resting muscle shear elastic modulus. Physiological measurement. 2012;33: N19 10.1088/0967-3334/33/3/N19 22370174

[19] Koo TK, Guo J-Y, Cohen JH, Parker KJ. Quantifying the passive stretching response of human tibialis anterior muscle using shear wave elastography. Clinical Biomechanics. 2014;29: 33–39. 10.1016/j.clinbiomech.2013.11.009 24295566

[20] Dubois G, Kheireddine W, Vergari C, Bonneau D, Thoreux P, Rouch P, et al Reliable protocol for shear wave elastography of lower limb muscles at rest and during passive stretching. Ultrasound Med Biol. Elsevier; 2015;41: 2284–2291. 10.1016/j.ultrasmedbio.2015.04.020 26129731

[21] Eriksson Crommert M, Lacourpaille L, Heales LJ, Tucker K, Hug F. Massage induces an immediate, albeit short-term, reduction in muscle stiffness. Scand J Med Sci Sports. 2014;25: e490–e496. 10.1111/sms.12341 25487283

[22] Akagi R, Takahashi H. Effect of a 5-week static stretching program on hardness of the gastrocnemius muscle. Scand J Med Sci Sports. 2014;24: 950–957. 10.1111/sms.12111 23944602

[23] Green MA, Sinkus R, Gandevia SC, Herbert RD, Bilston LE. Measuring changes in muscle stiffness after eccentric exercise using elastography. NMR in Biomedicine. 2012;25: 852–858. 10.1002/nbm.1801 22246866

[24] Guilhem G, Doguet V, Hauraix H, Lacourpaille L, Jubeau M, Nordez A, et al Muscle force loss and soreness subsequent to maximal eccentric contractions depend on the amount of fascicle strain in vivo. Acta Physiol (Oxf). 2016;217: 152–163. 10.1111/apha.12654 26786411

[25] Lacourpaille L, Nordez A, Hug F, Couturier A, Dibie C, Guilhem G. Time-course effect of exercise-induced muscle damage on localized muscle mechanical properties assessed using elastography. Acta Physiol (Oxf). 2014;211: 135–146. 10.1111/apha.12272 24602146

[26] Millet GP, Millet GY. Ultramarathon is an outstanding model for the study of adaptive responses to extreme load and stress. BMC Medicine. 2012;10: 77 10.1186/1741-7015-10-77 22812424 PMC3407019

[27] Clarkson PM, Hubal MJ. Exercise-induced muscle damage in humans. American journal of physical medicine \& rehabilitation. 2002;81: S52–S69. 12409811 10.1097/00002060-200211001-00007

[28] Guilhem G, Cornu C, Maffiuletti NA, GuéVel A. Neuromuscular Adaptations to Isoload versus Isokinetic Eccentric Resistance Training. Medicine & Science in Sports & Exercise. 2013;45: 326–335. 10.1249/MSS.0b013e31826e7066 22903139

[29] Héroux ME, Gandevia SC. Human muscle fatigue, eccentric damage and coherence in the EMG. Acta Physiol. 2013;208: 294–295. 10.1111/apha.12133 23746370

[30] Semmler A, Okulla T, Kaiser M, Seifert B, Heneka MT. Long-term neuromuscular sequelae of critical illness. Journal of Neurology. 2013;260: 151–157. 10.1007/s00415-012-6605-4 22820684

[31] Tanter M, Bercoff J, Athanasiou A, Deffieux T, Gennisson J-L, Montaldo G, et al Quantitative Assessment of Breast Lesion Viscoelasticity: Initial Clinical Results Using Supersonic Shear Imaging. Ultrasound in Medicine \& Biology. 2008;34: 1373–1386. 10.1016/j.ultrasmedbio.2008.02.002 18395961

[32] Catheline S, Gennisson JL, Delon G, Fink M, Sinkus R, Abouelkaram S, et al Measurement of viscoelastic properties of homogeneous soft solid using transient elastography: An inverse problem approach. The Journal of the Acoustical Society of America. 2004;116: 3734 10.1121/1.1815075 15658723

[33] Blazevich AJ. Effects of Physical Training and Detraining, Immobilisation, Growth and Aging on Human Fascicle Geometry. Sports Medicine (Auckland, NZ). 2006;36: 1003–1017. 10.2165/00007256-200636120-00002 17123325

[34] Gennisson JL, Rénier M, Catheline S, Barrière C, Bercoff J, Tanter M, et al Acoustoelasticity in soft solids: Assessment of the nonlinear shear modulus with the acoustic radiation force. The Journal of the Acoustical Society of America. 2007;122: 3211 10.1121/1.2793605 18247733

[35] Weismann C, Mayr C, Egger H, Auer A. Breast Sonography– 2D, 3D, 4D Ultrasound or Elastography. Breast Care. 2011;6: 98–103. 10.1159/000327504 21673819 PMC3104899

[36] Yamakoshi Y, Sato J, Sato T. Ultrasonic imaging of internal vibration of soft tissue under forced vibration. IEEE Trans Ultrason, Ferroelect, Freq Contr. 1990;37: 45–53. 10.1109/58.46969 18285015

[37] Atkinson G, Nevill AM. Statistical methods for assessing measurement error (reliability) in variables relevant to sports medicine. Sports medicine. 1998;26: 217–238. 9820922 10.2165/00007256-199826040-00002

[38] Rousson V, Gasser T, Seifert B. Assessing intrarater, interrater and test-retest reliability of continuous measurements. Statistics in Medicine. John Wiley & Sons, Ltd; 2002;21: 3431–3446. 10.1002/sim.1253 12407682

[39] Cohen J. Statistical Power Analysis for the Behavioral Sciences. L. Erlbaum Associates; 1988.

[40] McGraw KO, Wong SP. A common language effect size statistic. Psychological Bulletin. American Psychological Association; 1992;111: 361–365. 10.1037/0033-2909.111.2.361

[41] Lakens D. Calculating and reporting effect sizes to facilitate cumulative science: a practical primer for t-tests and ANOVAs. 2015;: 1–12. 10.3389/fpsyg.2013.00863/abstract PMC3840331 24324449

[42] Fulford J, Eston RG, Rowlands AV, Davies RC. Assessment of magnetic resonance techniques to measure muscle damage 24 h after eccentric exercise. Scand J Med Sci Sports. 2014;25: e28–e39. 10.1111/sms.12234 24738493

[43] Botanlioglu H, Kantarci F, Kaynak G, Unal Y, Ertan S, Aydingoz O, et al Shear wave elastography properties of vastus lateralis and vastus medialis obliquus muscles in normal subjects and female patients with patellofemoral pain syndrome. Skeletal Radiology. 2013;42: 659–666. 10.1007/s00256-012-1520-4 22996306

[44] Giovanelli N, Taboga P, Rejc E, Simunic B, Antonutto G, Lazzer S. Effects of an Uphill Marathon on Running Mechanics and Lower Limb Muscles Fatigue. IJSPP. 2015 10.1123/ijspp.2014-0602 26390075

[45] Davies CT, Thompson MW. Physiological responses to prolonged exercise in ultramarathon athletes. Journal of Applied Physiology (Bethesda, Md: 1985). 1986;61: 611–617. 10.1152/jappl.1986.61.2.611 3745051

[46] Radin EL. Role of muscles in protecting athletes from injury. Acta Medica Scandinavica Supplementum. 1986;711: 143–147. 3465203 10.1111/j.0954-6820.1986.tb08943.x

[47] Newham DJ, Jones DA, Clarkson PM. Repeated high-force eccentric exercise: effects on muscle pain and damage. Journal of Applied Physiology (Bethesda, Md: 1985). 1987;63: 1381–1386. 10.1152/jappl.1987.63.4.1381 3693172

[48] Clarkson PM, Nosaka K, Braun B. Muscle function after exercise-induced muscle damage and rapid adaptation. Medicine & Science in Sports & Exercise. 1992;24: 512–520. 1569847

[49] Howell JN, Chleboun G, Conatser R. Muscle stiffness, strength loss, swelling and soreness following exercise-induced injury in humans. The Journal of Physiology. 1993;464: 183–196. 8229798 10.1113/jphysiol.1993.sp019629 PMC1175380

[50] Saugy J, Place N, Millet GY, Degache F, Schena F, Millet GP. Alterations of Neuromuscular Function after the World's Most Challenging Mountain Ultra-Marathon. Hug F, editor. PLoS ONE. 2013;8: e65596 10.1371/journal.pone.0065596 23840345 PMC3694082

[51] Proske U, Morgan DL. Muscle damage from eccentric exercise: mechanism, mechanical signs, adaptation and clinical applications. The Journal of Physiology. 2001;537: 333–345. 11731568 10.1111/j.1469-7793.2001.00333.x PMC2278966

[52] Whitehead NP, Weerakkody NS, Gregory JE, Morgan DL, Proske U. Changes in passive tension of muscle in humans and animals after eccentric exercise. The Journal of Physiology. Blackwell Publishing; 2001;533: 593–604. 10.1111/j.1469-7793.2001.0593a.x 11389215 PMC2278643

[53] Green MA, Sinkus R, Gandevia SC, Herbert RD, Bilston LE. Measuring changes in muscle stiffness after eccentric exercise using elastography: MEASURING MUSCLE STIFFNESS CHANGES AFTER ECCENTRIC EXERCISE USING MRE. NMR in Biomedicine. 2012;25: 852–858. 10.1002/nbm.1801 22246866

[54] Warren GL, Palubinskas LE. Human and animal experimental muscle injury models. Skeletal muscle …; 2008.

[55] Jamart C, Benoit N, Raymackers J-M, Kim HJ, Kim CK, Francaux M. Autophagy-related and autophagy-regulatory genes are induced in human muscle after ultraendurance exercise. Eur J Appl Physiol. Springer-Verlag; 2011;112: 3173–3177. 10.1007/s00421-011-2287-3 22194006

[56] Balnave CD, Allen DG. The effect of muscle length on intracellular calcium and force in single fibres from mouse skeletal muscle. The Journal of Physiology. Wiley-Blackwell; 1996;492 (Pt 3): 705–713. 8734983 10.1113/jphysiol.1996.sp021339 PMC1158893

[57] Yanagisawa O, Niitsu M, Kurihara T, Fukubayashi T. Evaluation of human muscle hardness after dynamic exercise with ultrasound real-time tissue elastography: A feasibility study. Clinical Radiology. 2011;66: 815–819. 10.1016/j.crad.2011.03.012 21529793

[58] Knechtle B, Duff B, Schulze I, Kohler G. A multi-stage ultra-endurance run over 1,200 km leads to a continuous accumulation of total body water. Journal of sports science \& medicine. 2008;7: 357. 24149903 PMC3761892

[59] Proske U, Morgan DL. Muscle damage from eccentric exercise: mechanism, mechanical signs, adaptation and clinical applications. The Journal of Physiology. 2001;537: 333–345. 11731568 10.1111/j.1469-7793.2001.00333.x PMC2278966

[60] Fridén J, Lieber RL. Eccentric exercise-induced injuries to contractile and cytoskeletal muscle fibre components. Acta Physiologica Scandinavica. 2001;171: 321–326. 11412144 10.1046/j.1365-201x.2001.00834.x

[61] Fridén J, Sjöström M, Ekblom B. A morphological study of delayed muscle soreness. Experientia. 1981;37: 506–507. 7250326 10.1007/BF01986165

[62] Millet GY, Tomazin K, Verges S, Vincent C, Bonnefoy R, Boisson R-C, et al Neuromuscular Consequences of an Extreme Mountain Ultra-Marathon. Tarnopolsky M, editor. PLoS ONE. 2011;6: e17059 10.1371/journal.pone.0017059 21364944 PMC3043077

[63] Kjaer M, Magnusson P, Krogsgaard M, Boysen Møller J, Olesen J, Heinemeier K, et al Extracellular matrix adaptation of tendon and skeletal muscle to exercise. J Anat. Blackwell Publishing Ltd; 2006;208: 445–450. 10.1111/j.1469-7580.2006.00549.x 16637870 PMC2100210

[64] Hjorth M, Norheim F, Meen AJ, Pourteymour S, Lee S, Holen T, et al The effect of acute and long‐term physical activity on extracellular matrix and serglycin in human skeletal muscle. Physiol Rep. 2015;3: e12473–19. 10.14814/phy2.12473 26290530 PMC4562559

[65] de Lange Pieter, Moreno M, Silvestri E, Lombardi A, Goglia F, Lanni A. Fuel economy in food-deprived skeletal muscle: signaling pathways and regulatory mechanisms. The FASEB Journal. 2007;21: 3431–3441. 10.1096/fj.07-8527rev 17595346

[66] Sahlin K, Shabalina IG, Mattsson CM, Bakkman L, Fernstrom M, Rozhdestvenskaya Z, et al Ultraendurance exercise increases the production of reactive oxygen species in isolated mitochondria from human skeletal muscle. Journal of Applied Physiology (Bethesda, Md: 1985). 2010;108: 780–787. 10.1152/japplphysiol.00966.2009 PMC2853199 20110545

[67] Bell RAV, Al-Khalaf M, Megeney LA. The beneficial role of proteolysis in skeletal muscle growth and stress adaptation. Skeletal Muscle. Skeletal Muscle; 2016;: 1–13. doi: 10.1186/s13395-016-0086-6 27054028 PMC4822268

[68] Overgaard K, Lindstrøm T, Ingemann-Hansen T, Clausen T. Membrane leakage and increased content of Na+ -K+ pumps and Ca2+ in human muscle after a 100-km run. Journal of Applied Physiology (Bethesda, Md: 1985). American Physiological Society; 2002;92: 1891–1898. 10.1152/japplphysiol.00669.2001 11960939

[69] SKENDERI KP, KAVOURAS SA, ANASTASIOU CA, YIANNAKOURIS N, MATALAS A-L. Exertional Rhabdomyolysis during a 246-km continuous running race. Medicine & Science in Sports & Exercise. 2006;38: 1054–1057. 10.1249/01.mss.0000222831.35897.5f 16775544

[70] Millet GY, Tomazin K, Verges S, Vincent C, Bonnefoy R, Boisson R-C, et al Neuromuscular Consequences of an Extreme Mountain Ultra-Marathon. Tarnopolsky M, editor. PLoS ONE. 2011;6: e17059 10.1371/journal.pone.0017059 21364944 PMC3043077

[71] Maufrais C, Millet GP, Schuster I, Rupp T, Nottin S. Progressive and biphasic cardiac responses during extreme mountain ultra-marathon. Am J Physiol Heart Circ Physiol. American Physiological Society; 2016;: ajpheart.00037.2016. 10.1152/ajpheart.00037.2016 26921434

[72] Kreider RB. Physiological considerations of ultraendurance performance. Int J Sport Nutr. 1991;1: 3–27. 1844400 10.1123/ijsn.1.1.3

[73] Neumayr G, Pfister R, Hoertnagl H, Mitterbauer G, Getzner W, Ulmer H, et al The effect of marathon cycling on renal function. Int J Sports Med. © Georg Thieme Verlag Stuttgart · New York; 2003;24: 131–137. 10.1055/s-2003-38205 12669260

[74] McCarthy DA, Dale MM. The leucocytosis of exercise. A review and model. Sports Medicine (Auckland, NZ). 1988;6: 333–363. 10.2165/00007256-198806060-00002 3068772

[75] Hikida RS, Staron RS, Hagerman FC, Sherman WM, Costill DL. Muscle fiber necrosis associated with human marathon runners. J Neurol Sci. 1983;59: 185–203. 6854349 10.1016/0022-510x(83)90037-0

[76] Stupka N, Lowther S, Chorneyko K, Bourgeois JM, Hogben C, Tarnopolsky MA. Gender differences in muscle inflammation after eccentric exercise. Journal of Applied Physiology (Bethesda, Md: 1985). 2000;89: 2325–2332. 10.1152/jappl.2000.89.6.2325 11090586

[77] Manfredi TG, Fielding RA, O'Reilly KP, Meredith CN, Lee HY, Evans WJ. Plasma creatine kinase activity and exercise-induced muscle damage in older men. Medicine & Science in Sports & Exercise. 1991;23: 1028–1034. 1943622

[78] Roth SM, Martel GF, Ivey FM, Lemmer JT, Tracy BL, Hurlbut DE, et al Ultrastructural muscle damage in young vs. older men after high-volume, heavy-resistance strength training. Journal of Applied Physiology (Bethesda, Md: 1985). 1999;86: 1833–1840. 10.1152/jappl.1999.86.6.1833 10368346

[79] Newton MJ, Morgan GT, Sacco P, Chapman DW, Nosaka K. Comparison of responses to strenuous eccentric exercise of the elbow flexors between resistance-trained and untrained men. J Strength Cond Res. 2008;22: 597–607. 10.1519/JSC.0b013e3181660003 18550979

[80] Kim J, Lee J. The relationship of creatine kinase variability with body composition and muscle damage markers following eccentric muscle contractions. Journal of Exercise Nutrition and Biochemistry. 2015;19: 123–129. 10.5717/jenb.2015.15061910 26244131 PMC4523802

[81] Regueme SC, Nicol C, Barth lemy JL, Gr lot L. Acute and delayed neuromuscular adjustments of the triceps surae muscle group to exhaustive stretch-shortening cycle fatigue. Eur J Appl Physiol. 2004;93: 398–410. 10.1007/s00421-004-1221-3 15480740

[82] Avela J, Kyröläinen H, Komi PV, Rama D. Reduced reflex sensitivity persists several days after long-lasting stretch-shortening cycle exercise. Journal of Applied Physiology. 1999;86: 1292–1300. 10194215 10.1152/jappl.1999.86.4.1292

[83] Bouillard K, Jubeau M, Nordez A, Hug F. Effect of vastus lateralis fatigue on load sharing between quadriceps femoris muscles during isometric knee extensions. Journal of Neurophysiology. 2014;111: 768–776. 10.1152/jn.00595.2013 24259546

[84] Vitiello D, Degache F, Saugy JJ, Place N, Schena F, Millet GP. The increase in hydric volume is associated to contractile impairment in the calf after the world's most extreme mountain ultra-marathon. Extreme Physiology \& Medicine. 2015;4: 18 10.1186/s13728-015-0037-6 26500765 PMC4618124

[85] Voloshina AS, Ferris DP. Biomechanics and energetics of running on uneven terrain. Journal of Experimental Biology. 2015;218: 711–719. 10.1242/jeb.106518 25617451

[86] Sloniger MA, Cureton KJ, Prior BM, Evans EM. Lower extremity muscle activation during horizontal and uphill running. Journal of Applied Physiology. 1997;83: 2073–2079. 9390983 10.1152/jappl.1997.83.6.2073

[87] Lee SSM, Spear S, Rymer WZ. Quantifying changes in material properties of stroke-impaired muscle. Clinical Biomechanics. 2015;30: 269–275. 10.1016/j.clinbiomech.2015.01.004 25638688 PMC7057856

[88] Hill JC, Millán IS. Validation of musculoskeletal ultrasound to assess and quantify muscle glycogen content. A novel approach. The Physician and Sportsmedicine. 2014;42: 45–52. 10.3810/psm.2014.09.2075 25295766

[89] Nieman DC, Shanely RA, Zwetsloot KA, Meaney MP, Farris GE. Ultrasonic assessment of exercise-induced change in skeletal muscle glycogen content. BMC Sports Sci Med Rehabil. 2015;7 10.1186/s13102-015-0003-z PMC4406335 25905021

