# Correction: Shear-Wave Elastography Assessments of Quadriceps Stiffness Changes prior to, during and after Prolonged Exercise: A Longitudinal Study during an Extreme Mountain Ultra-Marathon

**DOI:** 10.1371/journal.pone.0167668

**Published:** 2016-11-30

**Authors:** Pierre Andonian, Magalie Viallon, Caroline Le Goff, Charles de Bourguignon, Charline Tourel, Jérome Morel, Guido Giardini, Laurent Gergelé, Grégoire P. Millet, Pierre Croisille

In Table 1, the Sex (male/female) values under Finish and Recovery are incorrect. The values should be 27/0 and 27/0, respectively. Please see the corrected Table 1 here.

**Table 1:** Demographic and training profile data.

	Pre	Mid	Finish	Recovery
N	50	31	27	27
Sex (male/female)	46/4	31/1	27/0	27/0
Age (years)	43 ± 9.1	43 ± 8.6	43 ± 8.7	43 ± 8.6
Height (meters)	1.75 ± 6.2	1.75 ± 6.4	1.75 ± 6.3	1.75 ± 5.6
Weight (kg)	72.2 ± 8	71.7 ± 8.2	70.7 ± 7.2	70.8 ± 7.3
BMI (kg.m^-2^)	23.6 ± 2.0	23.4 ± 2.0	23.1 ± 2.1	23.1 ± 2.0
Body temperature (°C)	36.2 ± 0.9	37.3 ± 0.5	37.3 ± 0.5	37.1 ± 0.7
Pain	0.00 ± 0	4.1 ± 2.9	3.6 ± 2.9	1.08 ± 1.7
Training/week (n)	3.94 ± 1.7	3.94 ± 1.5	3.94 ±1.5	3.94 ± 1.5
Running experience (years)	14.2 ± 10.4	13.5 ± 10.5	13.3 ± 10.6	13.1 ± 9.8
Experience in ultramarathons (years)	5.3 ± 3.6	5.5 ± 3.6	5.6 ± 3.6	5.3 ± 3.6
Previous ultramarathons (n)	13 ± 10	11 ± 9	11 ± 9	11 ± 9
Limb dominance (R/L)	43/7	27/4	23/4	23/4

All values are presented as the mean (standard deviation)

Thigh pain was quantified on a visual analog scale

Body mass index (BMI) was calculated as weight/height squared (kg m^-2^)

Pre, Mid, Finish and Recovery were the four key measurement time points:

Pre (pre-race) measurements were performed within 4 days before the race

Mid (mid-race) measurements were performed at the mid-point of the race (148.7 km, D+9270 m)

Finish measurements were performed at the end of the race, within 1 h after finishing

Recovery measurements were performed after 48–72 h of recovery
